# Frequency of MRI-detected peripheral osteoarthritis in athletes during the Summer Olympics in Rio 2016

**DOI:** 10.1016/j.ocarto.2021.100199

**Published:** 2021-08-05

**Authors:** Alexander Merritt, Frank W. Roemer, Rafael Heiss, Mohamed Jarraya, Dorra Guermazi, Daichi Hayashi, Lars Engebretsen, Michel D. Crema, Ali Guermazi

**Affiliations:** a Department of Radiology, Boston University School of Medicine, Boston, MA, USA; b Department of Radiology, University of Erlangen-Nuremberg, Erlangen, Germany; c Department of Radiology, Massachusetts General Hospital, Harvard Medical School, Boston, MA, USA; d Department of Radiology, Stony Brook University, Renaissance School of Medicine, Stony Brook, NY, USA; e International Olympic Committee (IOC), Lausanne, Switzerland; f Institute of Sports Imaging, Department of Sports Medicine, French National Institute of Sports (INSEP), Paris, France; g Department of Radiology, U.S. Department of Veterans Affairs, Boston, MA, USA

**Keywords:** Osteoarthritis, Olympics, Athletes, MRI

## Abstract

**Objective:**

To describe the frequency and severity of magnetic resonance imaging (MRI) based peripheral osteoarthritis (OA) in athletes during the Rio de Janeiro 2016 Olympic Games.

**Methods:**

All MRIs of the peripheral joints in Olympic athletes, performed at the centralized imaging facility, either following acute trauma or for non-traumatic joint pain, were included. All MRIs were retrospectively reviewed for presence and severity of MRI-based OA using an adapted Outerbridge classification for cartilage and adapted classifications for other tissues. Scoring of MRI abnormalities was independently and retrospectively performed without reference to the on-site clinical reports. The frequencies of MRI-detected OA were tabulated and grouped into sports categories, athletes’ age (<25; 25–29; and ≥30 years of age), and sex.

**Results:**

11,274 athletes participated in the Games. 320 athletes underwent MRI of the peripheral joints. One hundred sixty (50.0%) were female, 109 (34.1%) were <25 years, 132 (41.3%) between the ages of 25 and 29 years old, and 79 (24.7%) ≥30 years old. 53 (16.6%) had MRI-based OA, with slightly more than half having severe OA. In every age category, severe OA was the most frequent finding and there was a linear trend for increased likelihood of having OA with increasing age (Cochran-Armitage test, p ​= ​0.009). Frequencies of OA were similar in male and female athletes. The wrist (29.2%) and the knee (23.3%) were the most commonly affected joints.

**Conclusions:**

MRI-defined OA was not uncommon among elite athletes in this selected sample.

## 1 Introduction

Osteoarthritis (OA) negatively affects elite athletes’ performance during their careers and is a burden affecting the quality of life of retired athletes. OA is associated with a higher level of morbidity and the number of years lived with some degree of disability is often substantial, especially in knee OA [1,2]. Retired athletes seem to be at higher risk for developing hip and knee OA compared to the general population [3], and a recent cross-sectional study showed considerable prevalence rates of knee (14.2%) and hip (11.1%) clinically diagnosed OA in retired Olympic athletes from Great Britain, with prevalence rates of knee and hip pain of 26.1% and 22.4% respectively [4]. Another study [5] reported a prevalence of lower extremity OA ranging from 16 to 95% in former elite athletes. Prevalence of hand OA in retired elite and recreational cricketers in England and Wales was reported to be 6% [6]. The early detection of OA features in joints from active elite athletes is crucial, as some specific adaptations and strategies could be applied and potentially change the progression rate of disease. To date, there is a paucity of literature regarding the frequency of OA in active Olympic athletes.

The 2016 Summer Olympics were hosted by the city of Rio de Janeiro, Brazil, from August 5th to 21st, 2016. A total of 11,274 athletes from 207 National Olympic Committees competed in 306 events across 28 sports [7]. MRI scans were available for all Olympic athletes during the games. Several prior publications have reported frequencies of various injuries (of different types and locations) sustained by Rio Olympic athletes [[8], [9], [10], [11], [12], [13], [14], [15], [16], [17], [18], [19]], however none of the published papers focused on the frequency of OA.

This study's aim was to retrospectively evaluate all MRIs obtained on athletes during the games for a variety of reasons to determine the frequency and severity of MRI-based OA in the main peripheral joints.

## 2 Methods

### 2.1 Athletes’ inclusion

All athletes who underwent an MRI of the following peripheral joints during the 2016 Summer Olympic games were included in this study: shoulder, elbow, wrist, hip, knee, and ankle. Athletes were imaged for a multitude of reasons but had an MRI mainly for suspected structural damage following acute trauma or non-traumatic joint pain. MRIs were performed at the Polyclinic located within the IOC Olympic Village using two MR scanners (3.0-T Discovery MR750w and 1.5-T Optima 450 MRw; GE Healthcare, Brazil). Imaging data were collected retrospectively from the Centricity Radiological Information System and Picture Archiving and Communication System, both of which were provided by GE Healthcare. These data were stratified according to sex, age, type of sport, and peripheral joint. The data remained confidential and were de-identified in the medical database after the games. Our study was approved by the medical research ethics committee of the South-Eastern Norway Regional Health Authority (S–07196C). Because the information in this study was anonymized and deidentified, the need for informed consent was waived. The International Olympic Committee granted approval to use the anonymized imaging and demographic data for publication. An additional institutional review board approval was obtained from Boston University (H-36593). The athletes’ information was collected, stored, and analyzed in strict compliance with data protection and confidentiality.

### 2.2 Data analysis

All MRI examinations were independently and retrospectively reviewed for presence and severity of OA features in each joint, primarily by one radiologist (MJ) with 5-years of experience and adjudicated by a second radiologist (AG) with 25-years of experience in musculoskeletal radiology. Scoring of MRI abnormalities was performed without reference to the on-site clinical reports issued at the time of imaging.

To define the presence of knee OA based on MRI, we used an MRI definition of knee OA based on a Delphi exercise including several experts in the field [5]. This definition requires the presence of a definitive osteophyte AND full thickness cartilage loss, OR one of the aforementioned features PLUS two or more minor features such as subchondral bone marrow lesion, partial thickness cartilage loss, bone attrition, and/or meniscal degeneration/maceration/subluxation.

To define the presence of hip and shoulder OA based on MRI, we used a slightly modified definition adhering to the concepts included in the MRI definition of knee OA [5] but considering intrasubstance signal changes or linear intralabral fluid-like signal communicating with the joint space of the glenoid or acetabular labrum to suggest degeneration or tear instead of the menisci.

Finally, to define the presence of OA in the remaining joints, we also used a modified definition adhering to the concepts previously described for the knee [5], excluding the meniscal component for the elbow and ankle, and considering intrasubstance degeneration or tear of the triangular fibrocartilage instead for wrists included.

Furthermore, for each joint, osteophytes were scored as present or absent. Cartilage damage was graded from 0 to 4 using a modified Outerbridge classification as: grade 0 ​= ​normal; grade 1 ​= ​signal changes and/or minor surface irregularities; grade 2 ​= ​cartilage thickness loss ≤50%; grade 3 ​= ​cartilage thickness loss more than 50% in depth but no full thickness damage; grade 4 ​= ​full thickness loss [20]. We did not consider grade 1 damage in this study, as the diagnostic performance of MRI in the detection of such lesions is poor [21,22].

We further categorized the severity of MRI-based OA in peripheral joints as follows: mild OA ​= ​presence of osteophyte and cartilage damage grade 2 (modified Outerbridge); moderate OA ​= ​presence of osteophyte and cartilage damage grade 3; severe OA ​= ​presence of osteophyte and cartilage damage grade 4.

Descriptive analysis of frequency of OA was performed for the entire sample of athletes that underwent an MRI of peripheral joints. We further categorized the frequencies of OA for athletes’ age (<25; 25–29; and ≥30 years of age), sex, and sports categories. Chi-square test was used to evaluate the overall association between age and OA, and that between gender and OA. Cochran-Armitage test was used to assess the linear trend between increasing age and likelihood of having OA.

## 3 Results

From the 11,274 athletes participating in the 2016 Olympic Games, 319 (2.8%) underwent MRI of one or more of the abovementioned peripheral joints. A total of 349 peripheral joints were examined utilizing MRI, with knees (N ​= ​124; 35.5%) and ankles (N ​= ​92; 26.4%) representing the most frequent joints examined in this sample. 13 athletes had bilateral joints imaged, including one athlete who also had a repeat imaging of one side of the joint, and 3 athletes who had the same joint imaged twice. 8 patients had two joints imaged at the same time. All of these were included in our analysis.

**Fig. 1:**
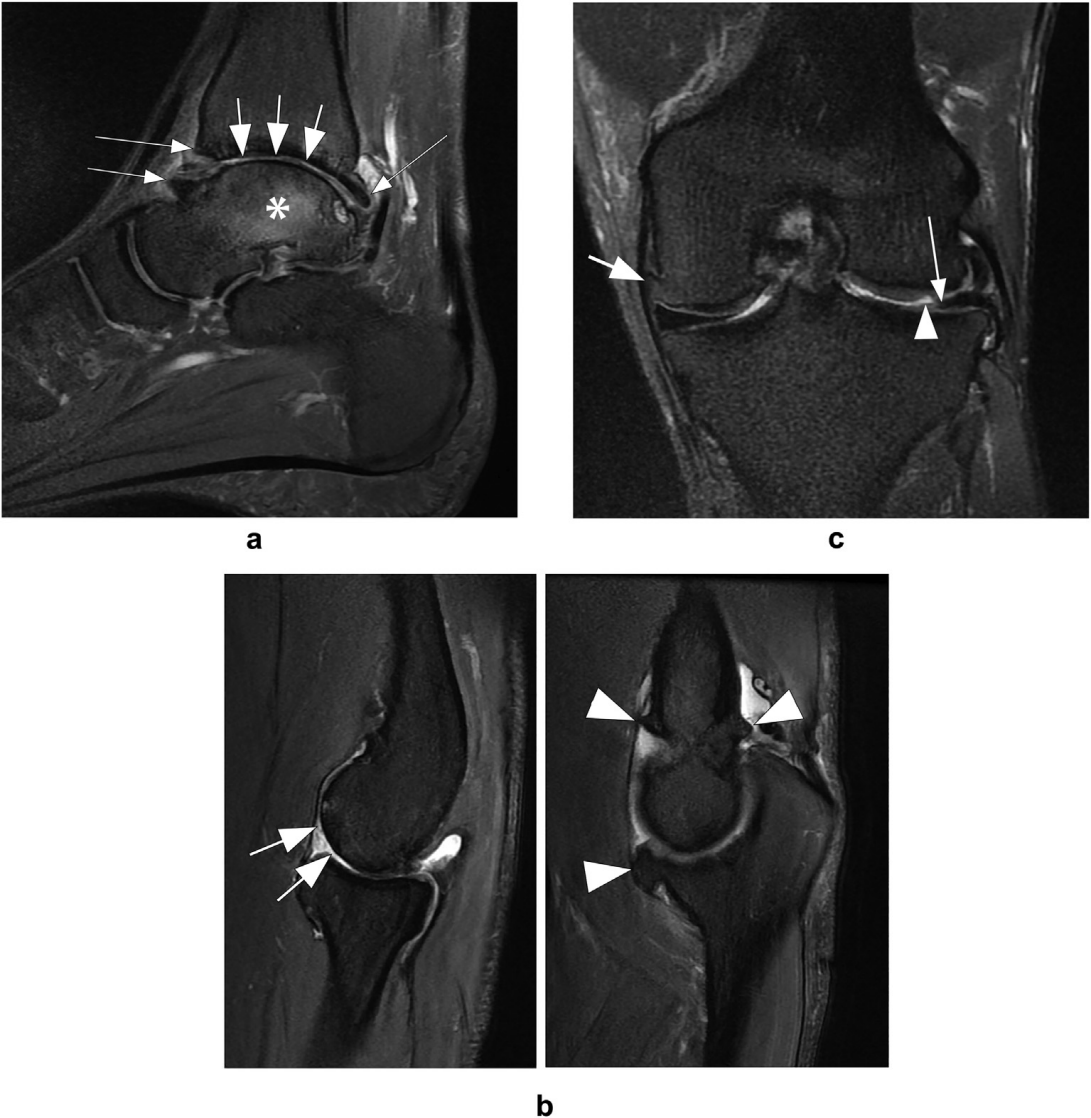
Example of different joints with osteoarthritis (OA) according to MRI assessment. A. Sagittal intermediate-weighted (IW) fat suppressed (fs) MRI of the ankle of a 34 year-old female athlete competing in track and field shows severe tibiotalar joint OA. There is diffuse wide-spread full thickness cartilage loss at the tibiotalar joint (short arrows). In addition there are definite anterior and posterior osteophytes (long arrows). Presence of these two features qualifies this joint for MRI-defined MRI-based OA. In addition there is marked subchondral bone marrow edema (asterisk) of the talus, a secondary feature of MRI OA. B. Sagittal IW fs images of the elbow of a 39 year-old male tennis player show diffuse full thickness cartilage loss at the capitellum humeri (arrows in the left image). In addition there are large marginal osteophytes (arrowheads in the right image). The combination of these two features qualifies this joint as having severe structural MRI OA. C. Coronal IW fs image of the knee in a 29 year-old weight lifter shows a definite marginal osteophyte at the medial femur (short arrow). In addition, there is a superficial focal cartilage defect (Outerbridge grade 2) at the lateral femur (arrowhead) and partial meniscal maceration of the free edge of the meniscal body (long arrow). Presence of these secondary features plus the presence of a definite osteophyte fulfills the criteria for a diagnosis of structural MRI OA. According to the definition used, this knee has mild OA.

The overall frequency of peripheral OA on a per athlete basis was 16.6% when considering the total number of athletes (53 out of 320), and 16% when considering the total number of peripheral joints examined on MRI (56 out of 349). Taking into account the total number of athletes having MRI-based OA, mild OA was found in 13 athletes (24.5% of 53 athletes), moderate OA in 13 (24.5%), and severe OA in 27 (50.9%). From all 56 joints exhibiting OA, the knee was the most common joint affected by OA (N ​= ​29; 54.7%), followed by the ankle (N ​= ​10; 18.9%) and the shoulder (N ​= ​7; 13.2%). Regarding age, MRI-detected peripheral OA was more common in athletes 30 years of age or older (22.8%) and was almost equally distributed in male and female athletes (18.1% and 15.0%, respectively). Fig. 1 shows examples of different joints with MRI-detected OA.

When considering each joint separately, the wrist (29.2%) and the knee (23.4%) were the most frequently affected by OA, with the knee (16.9%) and the shoulder (9.1%) exhibiting higher frequencies of severe OA (Table 1). When considering each age category, the frequencies of severe OA were similar for athletes between 25 and 29 years and athletes 30 years of age or older. Overall, there was no statistically significant association between age and OA (p ​= ​0.056) (Table 1). However, with increasing age, athletes were more likely to have OA (p ​= ​0.009). Frequencies of MRI-detected peripheral OA were similar between male and female athletes and there was no significant association between gender and OA (p ​= ​0.452) (Table 1).

**Table 1:** The upper half of the table shows the frequency and severity of MRI-detected OA by joint in the athletes who underwent MRI of peripheral joints during the Rio Olympics in 2016. N (%) ​= ​number and percentage of joints for a specific category. Total number of joints ​= ​349.

	No OA (%)	Any OA (%)	Mild OA (%)	Moderate OA (%)	Severe OA (%)
Joints					
Knee	95 (76.6)	29 (23.4)	0	8 (6.5)	21 (16.9)
Hip	39 (97.5)	1 (2.5)	1 (2.5)	0	0
Shoulder	48 (87.3)	7 (12.7)	0	2 (3.6)	5 (9.1)
Elbow	12 (85.7)	2 (14.2)	0	1 (7.1)	1 (7.1)
Ankle	82 (89.1)	10 (10.9)	10 (10.9)	0	0
Wrist	17 (70.8)	7 (29.2)	4 (16.7)	2 (8.3)	1 (4.2)
Total (Joints)	293 (84.0)	56 (16.0)	15 (4.3)	13 (3.7)	28 (8.0)
Age^a^^b^					
<25 years old	98 (89.9)	11 (10.1)	3 (2.8)	2 (1.8)	6 (5.5)
25–29 years old	108 (81.8)	24 (18.2)	5 (3.8)	6 (4.5)	13 (9.8)
≥30 years old	61 (77.2)	18 (22.8)	5 (6.3)	5 (6.3)	8 (10.1)
Gender^a^					
Male	131 (82.4)	29 (18.1)	7 (4.4)	6 (3.8)	15 (9.4)
Female	136 (84.4)	24 (15.0)	6 (3.8)	7 (4.4)	12 (7.5)
Total (athletes)	267 (85.0)	53 (16.6)	13 (4.1)	13 (4.1)	27 (8.4)

The lower half of the table shows the frequency and severity of OA by age and gender in the athletes who underwent an MRI of peripheral joints during the Rio Olympics in 2016. N (%) ​= ​number and percentage of athletes for a specific category. Total number of athletes ​= ​320.

a Based on chi-square test, the overall association between age and OA was not statistically significant (p ​= ​0.056), nor the association between gender and OA (p ​= ​0.452).

b Cochran-Armitage test for linear trend showed with age increase, it was more likely to have OA (p ​= ​0.009).

Regarding sports categories, peripheral OA was more frequently detected on MRI in athletes from gymnasts (N ​= ​10; 17.8%), followed by athletics performers (N ​= ​9; 16.1%), handball players and judo competitors (each exhibiting 5 OA cases; 8.9%) (Table 2). In all these sports, the knee was the most commonly joint affected by OA as detected on MRI.

**Table 2:** Distribution of OA in peripheral joints (total N ​= ​56) for each specific sport category.

Sport/Discipline	Knee	Hip	Shoulder	Elbow	Ankle	Wrist
**Archery**	0	0	0	0	0	0
**Athletics**	5 (8.9%)	0	2 (3.6%)	0	1 (1.8%)	1 (1.8%)
**Badminton**	1 (1.8%)	0	0	0	0	0
**Basketball**	1 (1.8%)	0	0	0	0	0
**Beach volleyball**	1 (1.8%)	0	0	0	2 (3.6%)	0
**Boxing**	0	0	1 (1.8%)	0	0	1 (1.8%)
**Cycling - Road**	0	0	0	0	0	1 (1.8%)
**Equestrian**	0	0	0	0	1 (1.8%)	0
**Gymnastics - Artistic**	2 (3.6%)	0	2 (3.6%)	0	5 (8.9%)	1 (1.8%)
**Gymnastics - Trampoline**	0	0	0	0	1 (1.8%)	0
**Handball**	5 (8.9%)	0	0	0	0	0
**Judo**	3 (5.4%)	1 (1.8%)	0	0	0	1 (1.8%)
**Rugby**	2 (3.6%)	0	1 (1.8%)	0	0	1 (1.8%)
**Tennis**	0	0	0	1 (1.8%)	0	0
**Taekwondo**	1 (1.8%)	0	0	0	0	0
**Volleyball**	2 (3.6%)	0	1 (1.8%)	0	0	0
**Weightlifting**	3 (5.4%)	0	0	0	0	1 (1.8%)
**Wrestling**	3 (5.4%)	0	0	1 (1.8%)	0	0

Joints with MRI detected OA were not present in the following sport disciplines: archery, cycling (mountain bike, track), canoe, aquatics (diving, open water swimming, swimming, synchronized swimming, water polo), football, fencing, golf, gymnastics (rhythmic), hockey, modern pentathlon, rowing, sailing, shooting, triathlon, table tennis.

## 4 Discussion

Our data demonstrates that athletes that underwent MRI of peripheral joints during the 2016 Rio de Janeiro Olympic Games had a frequency of OA of 16.6%. Out of these athletes with OA, the majority had severe OA. In addition, athletes with any OA were most frequently 30 years of age or older with a similar distribution in male and female athletes. MRI-detected severe OA was the most common feature of MRI-based OA in each age group, and its frequency was higher in male athletes, and those above 25 years of age. MRI-detected OA was more frequently found in the knee, which was one of the joints more commonly affected by severe OA in our sample. Peripheral joint OA was mainly detected in athletes from gymnastics and athletics, with the knee joint representing the main joint affected in these athletes.

The paucity of existing data on the frequency or prevalence of OA in active Olympic athletes limits the comparisons of our data with those from other sports populations. Most literature reported frequencies of OA in retired athletes [23,24]. A previous systematic review described the wide range of OA in former elite athletes, which stems from the fact that most studies used many different methods of assessing OA such as plain film, MRI, arthroscopy, or even self-reports [26]. Another study including former British Olympians aged 40 years or older found higher prevalence rates of knee and hip OA (14.2% and 11.1%, respectively) when compared with community populations [27]. This was based on questionnaires asking if these Olympians had a diagnosis of hip or knee OA by a physician with no imaging available to confirm nor to assess the severity of OA. From all knees assessed on MRI during the 2016 Summer Olympics, 23.4% had features of OA; this was true for only 2.5% of hips. Because the data we collected is not comparable and we did not examine all athletes participating in the Olympics, it is difficult to compare and discuss the frequencies found in these joints in our study with most available studies.

Given the high prevalence rates of peripheral OA in former athletes [[23], [24], [25], [26]], it is not surprising that most of the MRI-detected peripheral OA in the Olympic athletes was observed aged 30 years or older, including severe OA cases in our study. This reflects the repetitive stress and overload applied in peripheral joints, especially those from the inferior limbs when sports involve long-term weight-bearing activities. A previous study found an association between long-term weight-bearing sports and radiographic knee and hip OA in former middle-aged elite athletes [28].

Our study has several limitations. Firstly, observed frequency of MRI-depicted OA is dependent on the MRI-based definition of OA, and thresholds between different severity grades are somewhat arbitrary. Secondly, we assessed a highly selected sub-sample of Olympian athletes that presented for an MRI examination for a multitude of reasons. Clinical data was not available in this retrospective analysis and we do not know if these athletes had symptomatic OA. In addition, we did not obtain follow up information regarding the possibility of competing in the games, and if this was not possible, data on return to sport. Information such as duration of elite athletic activity or whether they were registered in prior Olympic competitions was not available. Future studies would benefit from reassessing the site of injury after a set period of recovery and/or the contralateral joint space. Clinical follow up — as well as assessment of future athletic achievements — would also increase the robustness of this study and should be considered in the future. Further limitation of our study includes lack of radiographic correlation, limited generalizability of our findings and potential selection bias, since we do not know the frequency of MRI-based OA in athletes who did not undergo MRI during Rio Olympic games.

In conclusion, we assessed a highly selected group of elite athletes who underwent MRI for one of six major joints during the 2016 Summer Olympics. These athletes were found to have a frequency of 16.6% of peripheral OA affecting mainly the knee joint. Clinical implications such as impact on athletes’ performance at competitions or overall career length remain to be determined by future studies with clinical correlation.

## Data statement

Research data used in our study is confidential and not available to public.

## Ethics statement

Our study was approved by the medical research ethics committee of the South-Eastern Norway Regional Health Authority (S–07196C). Because the information in this study was anonymized and deidentified, the need for informed consent was waived. The International Olympic Committee granted approval to use the anonymized imaging and demographic data for publication. An additional institutional review board approval was obtained from Boston University (H-36593). The athletes’ information was collected, stored, and analyzed in strict compliance with data protection and confidentiality.

## Authors contributions

Blinded for review. See CRediT statement.

## Declaration of competing interest

AG is also shareholder of BICL, LLC, and Consultant to Pfizer, Novartis, Regeneron, AstraZeneca, MerckSerono and TissueGene. FWR and MDC are shareholders of BICL, LLC. FWR is consultant to Calibr – California Institute of Biomedical Research. Other authors have nothing to disclose.
